# Long-Term Results of Kidney Transplantation in the Elderly: Comparison between Different Donor Settings

**DOI:** 10.3390/jcm10225308

**Published:** 2021-11-15

**Authors:** Renana Yemini, Ruth Rahamimov, Ronen Ghinea, Eytan Mor

**Affiliations:** 1 Department of Surgery, Samson Assuta Ashdod University Hospital, Ashdod 7747629, Israel; renanayemini@gmail.com; 2 Faculty of Health Sciences, Ben Gurion University of the Negev, Be’er Sheva 8410501, Israel; 3 Institute of Nephrology, Beilinson Medical Center, Petach-Tikva 49100, Israel; rutir@clalit.org.il; 4 Sackler Medical School, Tel-Aviv University, Tel-Aviv 6997801, Israel; rghinea@hotmail.com; 5 Transplant Unit, Department of Surgery B, Sheba Medical Center, Ramat Gan 52621, Israel

**Keywords:** dialysis, elderly, expanded criteria donor, kidney transplantation

## Abstract

With scarce organ supply, a selection of suitable elderly candidates for transplant is needed, as well as auditing the long-term outcomes after transplant. We conducted an observational cohort study among our patient cohort >60 years old with a long follow up. (1). Patients and Methods: We used our database to study the results after transplant for 593 patients >60 years old who underwent a transplant between 2000–2017. The outcome was compared between live donor (LD; n = 257) recipients, an old-to-old (OTO, n = 215) group using an extended criteria donor (ECD) kidney, and a young-to-old (YTO, n = 123) group using a standard-criteria donor. The Kaplan−Meir method was used to calculate the patient and graft survival and Cox regression analysis in order to find risk factors associated with death. (2). Results: The 5- and 10-year patient survival was significantly better in the LD group (92.7% and 66.9%) compared with the OTO group (73.3% and 42.8%) and YTO group (70.9% and 40.6%) (*p* < 0.0001). The 5- and 10-year graft survival rates were 90.3% and 68.5% (LD), 61.7% and 30.9% (OTO), and 64.1% and 39.9%, respectively (YTO group; *p* < 0.0001 between the LD and the two DD groups). There was no difference in outcome between patients in their 60’s and their 70’s. Factors associated with mortality included: age (HR-1.060), DM (HR-1.773), IHD (HR-1.510), and LD/DD (HR-2.865). (3). Conclusions: Our 17-years of experience seems to justify the rational of an old-to-old allocation policy in the elderly population. Live-donor transplant should be encouraged whenever possible. Each individual decision of elderly candidates for transplant should be based on the patient’s comorbidity and predicted life expectancy.

## 1. Introduction

Patients ≥60 years old are the largest growing age group in the end-stage renal disease (ESRD) population and comprise 40% of all patients with ESRD. About 20% of the candidates awaiting a transplant at any time in the given year are elderly patients [1]. The American Society of Transplantation evaluation guidelines state that there should be no absolute upper age limit for excluding patients whose overall health and life situation suggest that transplantation will be beneficial [2]. Kidney transplantation (KT) also offers a survival benefit in elderly patients, yet a subgroup analysis showed a diminished survival benefit in the 70–74 year-old age group [3]. Nevertheless, KT in the elderly population remains a controversial issue, especially among the age group of >70 years old. Rao et al. [4] showed the outcomes of 5667 elderly patients >70 years old waitlisted between the years 1990 and 2004. They found a 41% reduction in risk of death in patients transplanted versus patients who remained on the waiting list. They also showed that the recipients of expanded criteria donor kidneys had a 25% reduction in risk of death compared with patients who remained on the waiting list. The majority of patients in the elderly age group have associated comorbidities like diabetes, coronary artery disease, and peripheral vascular disease, which make them more frail and ineligible as transplant candidates [5,6]. However, certain elderly patients are good transplant candidates and have a significant survival benefit and improved quality of life after transplant. The question is what are the predictors of a good outcome, namely prolonged graft and patient survival, without associated posttransplant complications requiring readmissions? Previous reports have tried to delineate parameters that will help define this group of patients. In a report using decision analytic model comparing deceased donor KT to continued hemodialysis treatment, the authors concluded that if available within a timely period (<2 years), transplantation may offer substantial clinical benefits to older patients at a reasonable financial cost. Prolonged waiting times dramatically decrease the clinical benefits and economic attractiveness of transplantation [7]. In another large single center cohort study including 233 patients older than 65 years transplanted over a 15 year period, Heldal et al. showed that KT in these patients offered a survival advantage over dialysis treatment [8]. In their series of patients remaining on the transplantation waitlist, median survival from waitlisting was 3.4 (3.0–3.8) years compared with 4.8 (3.8–5.9) years in the transplant group. The 5-year survival of KT recipients was 66% compared with 33% among the waitlist patients. When looking at the scarce organ resource, the individual benefit of transplanting elderly patients has to be balanced against the corresponding increase in the number of patients awaiting grafts. In the above study from Finland, the median dialysis time for transplanted patients was only 12 months, reflecting a high organ donation rate, which justifies allocation of kidneys to elderly patients. This is not true however in many other parts of the world, where the median waiting time for transplant, such as in the US, can reach 4–5 years [1]. To overcome this limitation, several countries have created allocation policies that adjust the predicted recipient life expectancy with the projected graft survival by using extended criteria donor (ECD) in the elderly patients [9].

Eurotransplant Leiden started the Eurotransplant Senior Program “old for old” in 1999. Their allocation system placed a cut-off age of 65 for matching between donors and recipients. The kidneys are transplanted with a short cold ischemia time regardless of the human leukocyte antigen (HLA) compatibility [10]. In parallel, at also in 1999, the Israeli National Transplantation Center approved a similar “old-to-old” program for kidney transplantation using kidneys from donors >60 years old or ECD kidneys from donors >50 years old in patients older than 60 [11,12]. In the US, in order to implement a similar concept, a policy enabling the use of ECD kidneys was implemented in November 2000 [9,13]. In 2012, the US Organ Procurement and Transplantation Network (OPTN) replaced the ECD classification system with the Kidney Donor Profile Index (KDPI), which provided an estimate of the expected survival of a deceased donor kidney graft and a means to evaluate the suitability of deceased donor kidney possibilities. KDPI was calculated from donor variables including age, race, diabetes, hypertension, serum creatinine, height, weight, hepatitis C seropositivity, and cause of death, using the method described by the OPTN [14,15].

In this study, we report the long-term KT results at our center within the “old-to-old” program, and specifically analyze the outcomes in a subgroup of patients 70 years and older. A further analysis was done to define the risk factors associated with graft failure and patient death among our patient cohort older than 60 years old.

## 2. Patients and Methods

### 2.1. Patient Selection

This cohort study is based on a retrospective analysis of our center transplant database, including kidney transplants performed between 2000–2017. The study was approved by the Institutional Review Board of the Beilinson Medical Center. We used data of 593 kidney transplants in the elderly (aged >60 years) for the analysis.

First, we analyzed the long-term results of the subgroup of patients transplanted within the deceased donor old-to-old program (DD-OTO), including 213 patients, and compared them with two other groups, namely 123 patients who received a kidney from a deceased donor younger than 60 years old (DD-YTO) and another group of 257 patients who received a graft from a living donor (LD) during the same time interval. Then, we focused on the group of patients 70 and older and compared their graft and patient survival rates to that of patients in their 60s. Finally, we used a multifactorial regression analysis to find risk factors associated with graft loss and patient death in the elderly population.

Data were extracted from the medical records of the relevant hospital departments, including outpatient clinics, surgery, and anesthesia, and consisted of the recipient’s and donor’s age and sex, cause of ESRD (diabetic nephropathy, hypertensive disease, polycystic kidneys disease (PKD), focal and segmental glomerulosclerosis (FSGS), glomerulonephritis (GN), pyelonephritis, congenital, others, and unknown), preoperative weight and BMI (kg/m^2^), comorbidities (diabetes mellitus (DM), ischemic heart disease (IHD), and hypertension (HTN)), dialysis duration before transplantation, graft from an LD or DD, panel reactive antibody (PRA), and human leukocyte antigen (HLA)-DR mismatch (MM). Outcomes and complications were determined by analyses of the patients, who all had their follow-ups at our transplant center.

### 2.2. Deceased Donor Kidney Allocation

In the deceased donor old-to-old (DD-OTO) group, kidneys from donors >60 or ECD donors >50 years (defined as donors with at least two risk factors: history of diabetes mellitus or hypertension, serum Cr. >1.5 mg/dL, and CVA as a cause of death) were allocated to patients older than 60 years with a PRA of 0% on three consecutive recent samples. PRA 0% was defined until 2008 by the classical PRA serological test against 20 healthy controls, while after 2008, class I and class II HLA Ab’s was tested using Luminex technique (R&D System, Biotech Co., Minneapolis, MN, USA). In the deceased donor young-to-old (DD-YTO) group, allocation was based on the following four parameters: time on dialysis, degree of pre-sensitization according to percent PRA, B and DR-HLA matching, and age as a continuous variable. In 2012, two new parameters were added, namely (1) being a registered organ donor for over 3 years earned a patient two extra points, and (2) if a family member donated in the past, an extra nine points were added to the candidate’s score.

### 2.3. Operative Management

Kidney transplantation was performed through an extraperitoneal approach in the iliac fossa. The renal vessels were anastomosed to the external iliac vessels, and the ureter was implanted into the bladder by an extravesical uretero-cystostomy using the anti-reflux technique. A double-J stent was routinely placed in the ureter and was removed 3 to 6 weeks after transplantation.

### 2.4. Perioperative Management

Maintenance immunosuppression included the calcineurin inhibitors tacrolimus (Prograf, Astellas Pharma, Middlesex, UK) starting on postoperative day 1 at a dose of 0.15 mg/kg, target 12-h trough levels of 8 to 12 ng/mL during the first 3 months and 5 to 8 ng/mL thereafter, or cyclosporine (Sandimmune Neoral, Novartis Pharmaceutical) starting on postoperative day 1 at a dose of 8 mg/kg, target 12-h trough levels of 150 to 300 ng/mL during the first 3 months and 100 to 200 ng/mL thereafter. Antiproliferative agents included 1000 mg mycophenolate mofetil (Cellcept, Roche Pharmaceuticals) twice daily for the first 2 weeks and 500 mg three times a day thereafter, or mycophenolic acid (Myfortic, Novartis Pharma) 720 mg twice daily for the first 2 weeks and 360 mg three times per day thereafter. All patients received perioperative intravenous corticosteroid therapy with methylprednisolone 500 mg on day 0, 250 mg on day 1, and 100 mg on day 2, after which they received oral prednisone 20 mg per day tapered to 5 mg per day within 3 months. Induction therapy consisted of one of the following: the anti–IL-2 receptor antagonist basiliximab (Simulect, Novartis Pharma) administered intravenously on days 0 and 4 at a dosage of 20 mg; daclizumab (Zenapax; Roche Pharmaceuticals, Basel, Switzerland) at a dosage of 1 mg/kg on days 0 and 14; or, in cases of immunologic high risk, rabbit antithymocyte globulin (ATG) (Thymoglobulin; Genzyme Corp) at an intravenous dosage of 1.0 mg/kg daily for 3 days starting intraoperatively. Part of the study population did not receive induction therapy. In January 2014, we changed our induction protocol for low risk (non-sensitized) deceased-donor patients and instead of Basilixumab, we used a single dose of Thymoglobulin in the OR.

### 2.5. Clinical Outcomes

The primary clinical outcomes of this study were graft failure (defined as death or return to dialysis), death-censored graft failure, and all-cause mortality. The outcome data of all recipients were censored in August 2019. Secondary outcomes were delayed graft function (DGF) defined as one or more dialysis after transplantation and primary non- function (PNF). Length of hospital stay after transplant and graft function were measured by Cr levels immediately and long-term after transplant.

### 2.6. Statistical Analysis

Mean values, standard deviations, and absolute and relative frequencies were calculated for the descriptive statistical analysis. Chi-squared tests were used to assess the difference in the frequencies between the four groups for categorical variables, and t-tests and analysis of variance (ANOVA) were applied for continuous variables. Variables that were significant on the univariate analysis were entered into a multivariate analysis. *p* values ≤ 0.05 were considered significant. Survival analysis was by the Kaplan–Meir Method, with the log rank test d for comparisons between groups and the Cox regression analysis applied for identifying risk factors for graft loss and demise. The results were expressed as hazard ratio (HR) with 95% confidence interval (CI). The covariates included in the logistic regression and Cox regression models were donor age and gender, recipient age and gender, diabetes, ischemic heart disease, graft type, dialysis (yes/no) prior to transplant, sensitization (PRA > 10%), and re-transplantation. Effect modification between donor types with covariates and outcomes were also examined. Variables that had an association with clinical outcomes with *p*-values of <0.10 in the unadjusted analyses were included in the multivariable-adjusted analyses. Statistical analyses were performed by IBM SPSS Statistics, software version 25.0 (IBM Corp., Armonk, NY, USA).

## 3. Results

### 3.1. Comparison between the Live Donor Group (LD) and the Two Deceased Donor Groups: Old-to-Old (DD-OTO) and Young-to-Old (DD-YTO) Recipients

**Figure 1:**
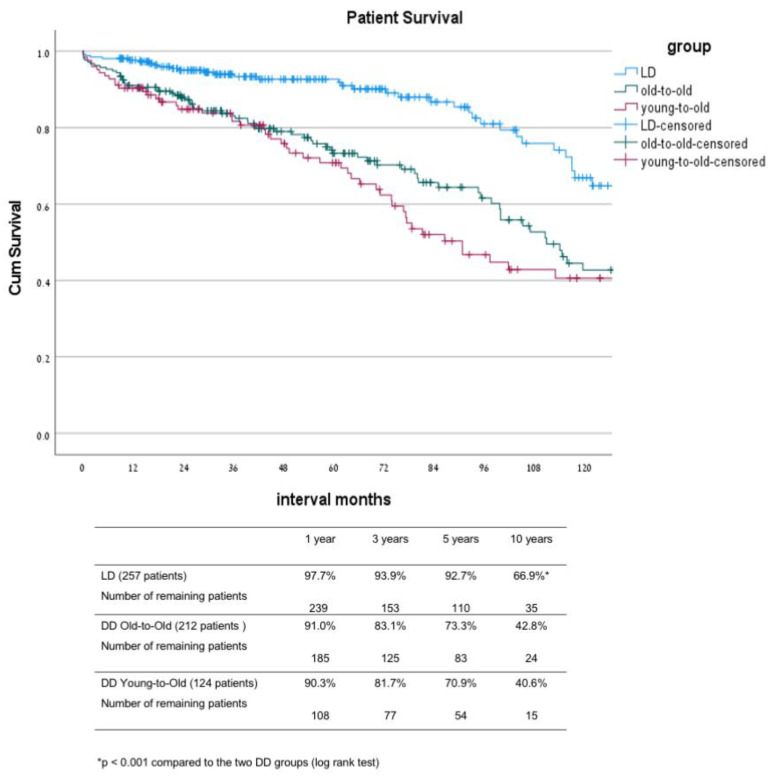
Kaplan–Meier patient survival of LD, DD-OTO, and DD-YTO. DD, deceased donor; LD, living donor; OTO, old-to-old; YTO, Young-to-Old.

**Figure 2:**
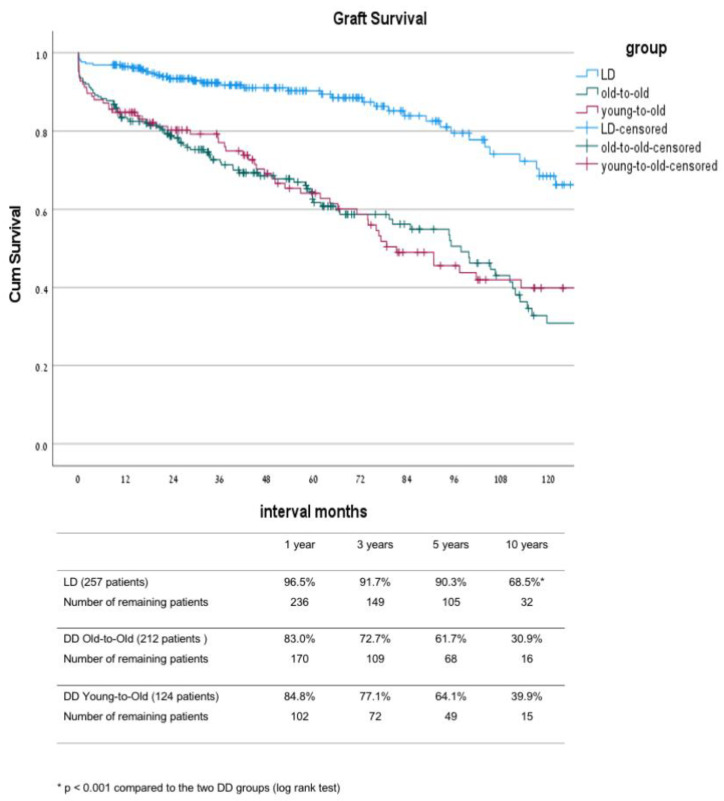
Kaplan–Meier graft survival of LD, DD-OTO, and DD-YTO. DD, deceased donor; LD, living donor; OTO, old-to-old; YTO, Young-to-Old.

**Figure 3:**
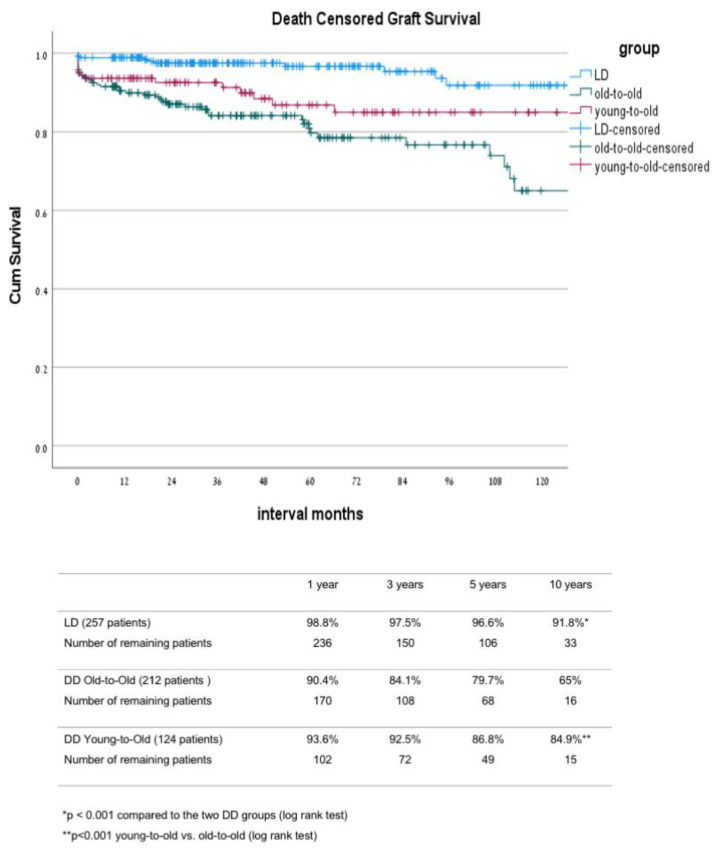
Kaplan–Meier death censored graft survival of LD, DD-OTO, and DD-YTO. DD, deceased donor; LD, Living donor; OTO, old-to-old; YTO, Young-to-Old.

In comparison between the three groups, the main differences were noted between the LD group and the two DD groups (Table 1). As part of the allocation, differences between these groups were that DD-OTO patients were non-sensitized with a lower level of HLA matching and a lower rate of re-transplantation. Their donor’s mean age was significantly higher compared with the age of the donors in the two other groups. The LD patients had a shorter duration of dialysis before transplant, with 25% of them transplanted before the initiation of dialysis. The induction protocol was also different between the LD and the two DD groups, with a greater proportion of patients in the DD groups who received a single dose of ATG instead of IL-2 inhibitors as part of a new protocol introduced in 2013. The mean donor age of the LD and the DD-YTO groups was significantly lower compared to the mean donor age of the DD-OTO group. The above differences were translated into a better outcome in the LD group compared to that of the other two DD groups, including significantly lower DGF and PNF rates (Table 2 and Table 3) as well as better graft and patient survival rates (Figure 1 and Figure 2). The ten year uncensored patient survival rates were 42.8% in deceased donor old-to-old (DD-OTO), 40.6% in deceased donor young-to-old (DD-YTO), and 66.9% in live donor (LD), *p* = 0.000 LD compared to the two DD groups. The ten year uncensored graft survival rates were 30.9% in DD-OTO, 39.9% in DD-YTO, and 68.5% in LD, *p* = 0.000 LD, compared to the two DD groups (Figure 1 and Figure 2). Graft and patient survival were similar in the two DD groups (Figure 1 and Figure 2). Recipients in the LD and DD-YTO groups had a better death-censored graft survival (Figure 3), although graft and patient survivals were not different between the two DD groups. The estimated 10 year graft survival censored for death with a functioning graft was 65% in DD-OTO, 84.9% in DD-YTO, and 91.8% in LD, *p* = 0.000 LD, compared to the two DD groups and *p* < 0.001 DD-YTO vs. DD-OTO (Figure 3).

In the Cox regression analysis (Table 3) looking for independent variables associated with risk of death after transplant, the following risk factors were found: age (HR 1.060), DM (HR 1.773), IHD (1.510), and donor type (DD vs. LD, HR 2.865). Variables associated with a risk of graft loss were IHD (HR 1.782), donor age (HR 1.025), and donor type (DD vs. LD, HR 6.064).

### 3.2. Comparison between Patients 70 and Older to Patients 60–69 Years

In the second part of our study, we compared the results after kidney transplantation in a subgroup of recipients 70 years and older (*n* = 100) to the remaining cohort of 60–69 year old patients (*n* = 493). Apart from the differences in mean age, the mean donor age and proportion of male to female were both higher in the patients ≥70 years old. Other parameters, including primary disease proportion of patients with DM and IHD, degree of sensitization, interval of dialysis pretransplant, HLA DR match, donor gender, induction type, and re-transplant rate, were not significantly different between the two groups. Living donor rates were lower in the ≥70 year old patient group with 12.8% compared to 19.8% in the 60–69 year old group (*p* = 0.016) (Table 4).

**Figure 4:**
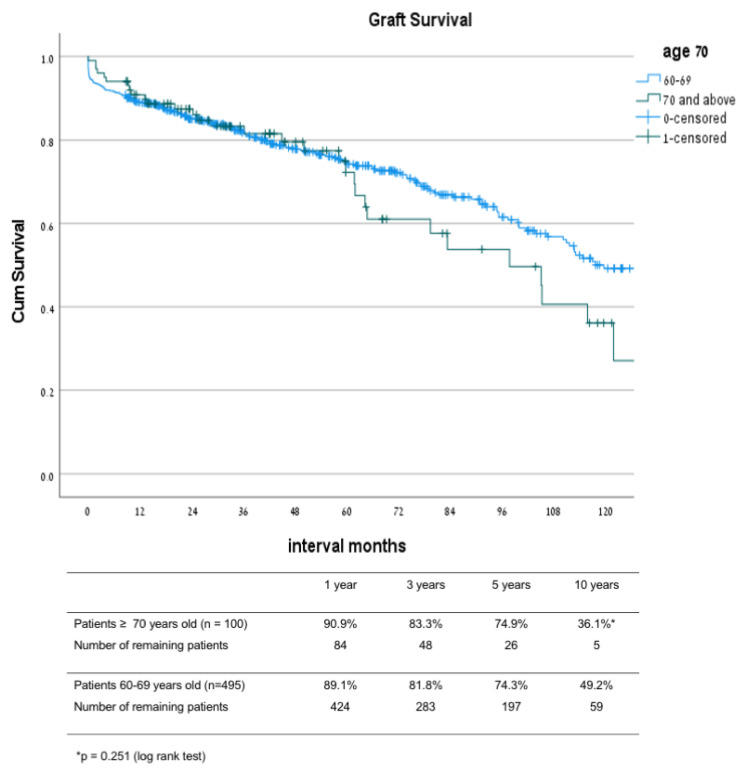
Graft survival: comparison between groups 60–69 and ≥70 years old.

**Figure 5:**
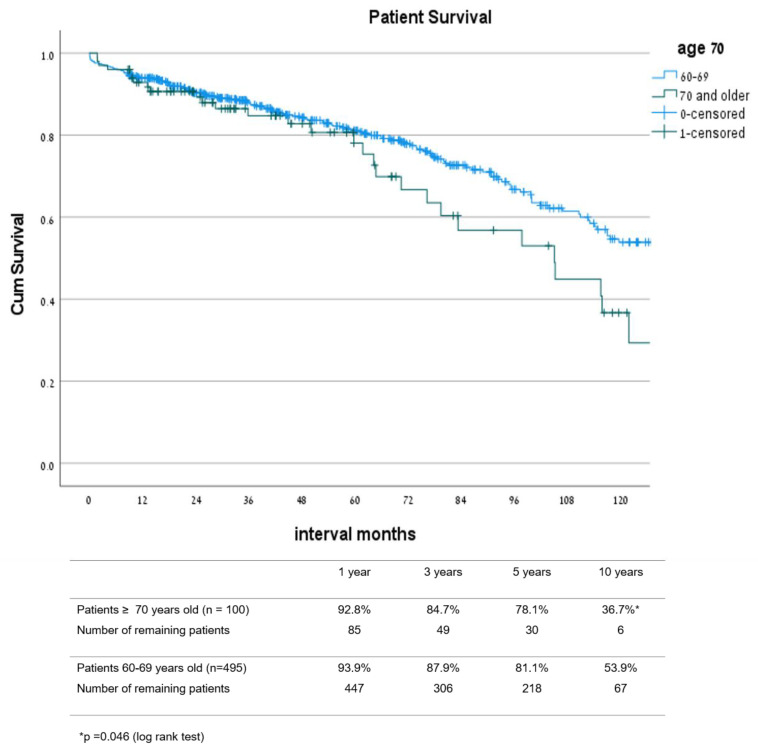
Patient survival: comparison between groups 60–69 and ≥70 years old.

**Figure 6:**
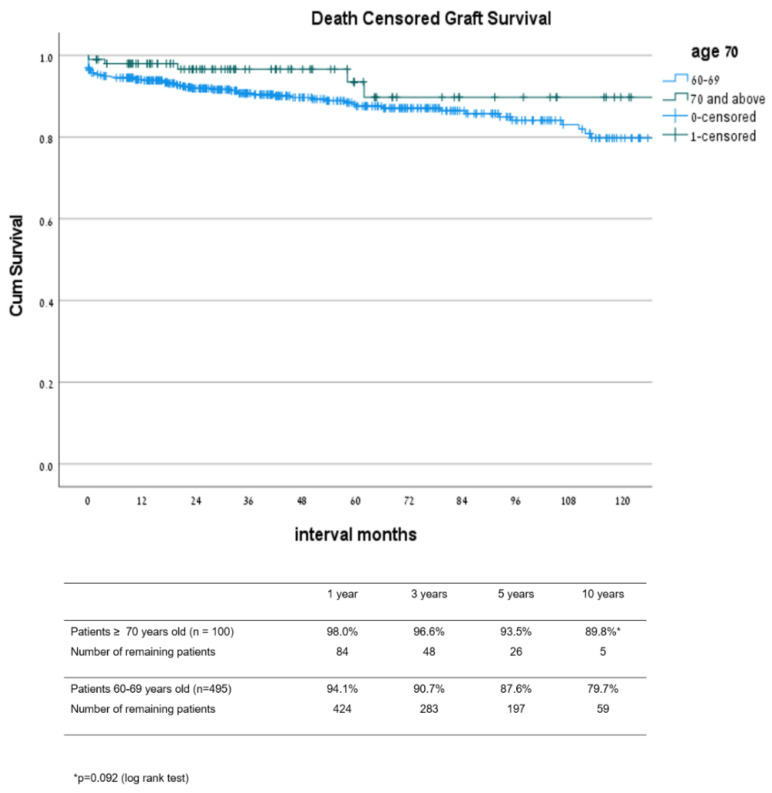
Estimated graft survival censored in death with functioning graft: comparison between groups 60–69 and ≥70 years old.

Graft survival rates (Figure 4) at 1, 3, 5, and 10 years after transplant in patients 70 and older were 90.9%, 83.3%, 74.9%, and 36.1%, respectively, while in patients 60–69 years old, the survival rates were 89.1%, 81.8%, 74.3%, and 49.2% (*p* = 0.251), respectively. Patient survival rates (Figure 5) at 1, 3, 5, and 10 years after transplant were 92.8%, 84.7%, 78.1%, and 36.7% in the 70 and older group, and 93.9%, 87.9%, 81.1%, and 53.9% in the 60–69 year old group, respectively (*p* = 0.046). Estimated graft survival censored in death with functioning graft (Figure 6) at 1, 3, 5, and 10 years after transplant were 98.0%, 96.6%, 93.5%, and 89.8% in the ≥70 year old group compared to 94.1%, 90.7%, 87.6%, and 79.7% in the 60–69 years old group, *p* = 0.092.

The rates of DGF, PNF, overall graft failure, death rates, length of hospital stay, and mean Cr levels at 1 month, 1 year, and 5 years after transplant were not different between the two groups (Table 5).

## 4. Discussion

It is well documented that kidney transplantation offers a survival benefit in the elderly population when compared to dialysis, and that chronological age should not be a barrier for access to transplant [16]. In our large cohort study with a long follow up, a median of 5 years, the 5-year survival rates for live donor recipients were of 92.7% and for deceased donor recipients >70%, which were better than the expected survival if remaining on dialysis. In our group of patients older than 70 years, the 3- and 5-survival rates were 84.7% and 78.1%, respectively, and were slightly lower than those for patients a decade younger. However, in our multivariate Cox regression analysis, age remained an important factor affecting survival, as expected with increased risk of death with a functioning graft in advanced age, as seen in our study. Kidney transplantation from deceased donors >65 or older is associated with suboptimal patient and graft survival. Nevertheless, when compared to patients remaining on dialysis there is reduced risk for death [17]. In a study published by Loveras et al., a paired survival analysis between recipients of kidneys from DD older than 65 with that of their paired patients on maintenance dialysis. They observed that patient and graft survival was reduced with the increasing age of the donor and recipient. Moreover, elderly recipients of these old kidneys had a reduced risk of death-censored graft failure compared to younger recipients of these grafts [18]. On the other hand, it has been reported that old recipients of young donor kidneys show graft survival exceeding patient survival, which means a significant graft-years loss [19]. 

Historically, there has been reluctance to use living donor transplants for older adults given their inherent limited life span. However, recent data suggest that living donor kidneys might be the best treatment option for elderly transplant recipients, just as it is for younger individuals [20]. Molnar et al. compared the association of ECD kidney and living kidney donation across different ages, including elderly recipients. They concluded that living donor kidney appears to be associated with greater survival across all age groups, including the elderly, although the significantly lower transplant loss rate is observed mainly in those younger than 70 years. Hence, they suggested that the elderly patients with ESRD gain years of life if they receive a kidney transplant, in particular from a living donor. Other data indicate that elderly transplant recipients have a 41% lower overall risk of death compared with wait-listed candidates [21]. Kidney donation from the patient’s children in this age group may often be the only option, although it is not always accepted by the parent. Alternatively, a kidney donation from an older donor of the same age group should be considered. There are reports of good outcomes when using a graft from elderly living donors [22,23]. Given the relatively high probability of a poor outcome for older patients on the wait list, living donor transplantation, even with a donor 65 years or older, is preferable to waiting for a standard criteria deceased donor transplant [24]. Similar to previous reports in our study when comparing the outcome between three groups, live-donor and two groups of deceased donors also found a significant better patient and graft survival in the group of live donor recipients. These differences could be explained by the younger donor age, shorter duration of dialysis, and shorter cold ischemia in that group. Indeed, a Cox regression analysis including all these variables the type of donor remained the most significant variable affecting survival with HR of 2.865 (CI 1.910–4.297) for patient survival and 6.064 (CI 2.315–15.881) for graft survival. This finding reflects a better graft quality in the LD group when compared to the quality of grafts of the two DD groups associated with significantly lower rates of DGF and PNF after transplant, as well as lower creatinine levels along the follow up.

Differences between these two groups might also be contributed to by the selection bias of recipients with a better condition. In our study, 25% of patients in the LD group were transplanted before initiation of dialysis, whereas the mean dialysis span between initiation of dialysis to transplant in the two DD groups was >5 years. It is known that the longer elderly patients are on dialysis the worse is their general condition and frailty score, mainly because of the progression of cardiovascular and metabolic bone disease [5]. Schold et al. showed that when accepting an ECD for an elderly patient who is more than 2 years old on dialysis, the survival benefit of transplant over dialysis is markedly lower [25].

In our study, cardiovascular disease was found to be a significant risk factor affecting both patient and graft survival after transplant. The hazard ratio for death was 1.773 (CI 1.241–2.532) and for graft loss 1.782 (CI 1.045–3.038). Similar findings were reported in a series from Brazil including 366 patients older than 60, where diabetes mellitus as a cause of renal failure had a hazard ratio (HR) of 1.507 (CI 1.038 to 2.189) for death after transplant [26]. Patient and graft survivals at 5 years in this series were 76.6% and 72.2% similar to the results in our series. The incidence of major cardiac events is maximal during the first month after transplantation, associated with the stress of surgery, fluid resuscitation and infectious complications associated with hemodynamic instability. To address that problem and lower the cardiovascular risk, there is a need to prepare those candidates with known IHD and to manage them in collaboration with a cardiologist familiar with transplantation. Revascularization of asymptomatic IHD does not reduce risks of mortality for people with type 2 diabetes nor in those undergoing major vascular surgery [27,28]. There are no contemporary data in ESRD to determine whether revascularization is helpful or harmful overall; however, the risks of revascularization in ESRD are higher than for the general population. Given these uncertainties, there is a recommendation for screening candidates at high risk for IHD at time of evaluation, in order to guide medical management and inform risk [29,30].

In our series, 29.7% of the patients had diabetic nephropathy as the cause for ESRD and their overall death rate along the follow up was 32.4% compared to 21.8% in the remaining cohort. Moreover, DM was associated with HR of 1.773 for death after transplant. Indeed, previous studies have also shown that diabetes mellitus is associated with lower graft and patient survival after transplant in the elderly population. The rationale behind allocation programs based on age matching between donors and elderly recipients was based on utility considerations. Despite lower survival rates when using ECD donors for that population, the survival advantage benefit over wait-list patients has been shown in previous reports. In a report of 244 patients transplanted along the two decades of the ESP program, patient survival rates at 1, 5, and 10 years were 91.7%, 66.3% and 38%, respectively. Death censored graft survival for the same intervals were 93.3%, 82.6%, and 70.4%, respectively [31]. In the US, where allocation system is based on match between ECD kidneys having a high KDPI and patient risk score, a gain in survival over staying on dialysis is seen when ECD kidneys with high KDPI are transplanted in high-risk patients [32]. In a study to predict survival after transplant the authors combined two scores, the estimated patient post-transplant survival (EPTS) score and KDPI score. An Estimated Post Transplant Survival (EPTS) score is assigned to all adult candidates on the kidney waiting list and is based on four factors: time on dialysis, current diagnosis of diabetes, prior solid organ transplants, and age. The score is associated with how long the candidate will need a functioning kidney transplant when compared with other candidates [33].As for candidates with an EPTS score of 80, 5-year waitlist survival was 47.6%, and 5-year post-KT survival was 78.9% after receiving kidneys with a KDPI of 20% and was 70.7% after receiving kidneys with a KDPI of 80%. The impact of KDPI on survival benefit varied greatly by EPTS score. For candidates with low EPTS scores (e.g, 40), survival benefit decreased with a higher KDPI but was still substantial even with a KDPI of 100% (>16 percentage points) [34].

In our study, the group of “old-to-old” had a patient survival of 1, 5, and 10 years of 91.0%, 73.3%, and 42.8%, respectively. Death censored graft survival rates in the same intervals were 90.4%, 79.7%, and 65.0%, reflecting comparable results of the ESP program. When evaluating the results in our patient population of 70 years and older, patient survival at 1- and 5- years were no different from the younger cohort, with the drop to 36.7% at 10 years, which is explained by their death for aging and associated comorbidities. No difference in graft survival was seen between these two groups. This is despite a younger donor age and in the 60–69 year old group (52.4 ± 13.8 and 57.8 ± 12.2, respectively, *p* < 0.0001) and a lower proportion of live donor. Yet, on the long term, about 30% of these patients are dying with a functioning graft, a death that is associated mostly with their cardiovascular comorbidities [29].

In a study calculating the costs of transplant in the elderly population assuming a 2-year wait-listed time, transplantation remained economically attractive for 70 years old patients incremental cost effectiveness (ICE), $79,359 per quality-adjusted life years (QALY), but was less economically attractive for those over 75 years of age (ICE, $99,553) or for 70 years old with either cardiovascular disease or diabetes (ICE, $126,751 and $161,090 per QALY, respectively) [7]. The authors concluded that transplantation compared with dialysis continues to increase life expectancy at an advanced age, but it does so at an increased cost. The data also show that for the older patients, the attractiveness of transplantation is highly sensitive to the time spent waiting for the transplant.

In our series, elderly patients who received DD kidney had a high rate of delayed graft function (~40%) requiring dialysis and a long mean hospital stay (16 days). Although the DGF rate was significantly lower in the LD group (9.7%), the length of mean hospital stay was still relatively long (12.5 days), which explains the higher costs associated of transplantation in that age group. Nevertheless, LD kidney transplantation even when using a match-age donor in our study, as well as in other previous reports, has shown favorable outcomes associated with prolonged survival and therefore should be advocated whenever possible.

The strength of our study is its large cohort of elderly patients with a long follow-up with a mean duration of 68 months. Moreover, this is a single center study with a uniform recipient and immunosuppressive protocol with minimal changes overtime and a steady donor screening. In addition, all patients had their follow-up throughout the whole period at our nephrology clinic.

However, our study has several limitations that bear mention. First, it is retrospective in design. Second, there are some missing data parameters such as dialysis interval prior to transplant and rejection episodes. There is a selection bias with living donor candidates being carefully selected immediately before their elective transplant, while DD transplants are urgent procedures. Annual evaluation of DD KT candidates enables the deterioration of existing comorbidities or the increase of new medical problems. As a consequence, some of the urgent DD KT are done under sub optimal conditions rather than aborting after years on the waitlist. The differences between LD and DD transplants makes them unmatched. Lastly, we did not use a frailty score in our study, a factor that is well known to influence outcome after transplant in the elderly population [5].

In summary, in our study of KT in elderly patients >60 years with a mean follow up of 5.6 years, we showed comparable results of the “old-to-old” program to those reported in the literature. In the whole patient cohort, patient survival was independent of donor age, while recipient age and comorbidities were significant factors affecting outcome in that age group. Donor age was a risk factor of graft loss with HR of 1.025 (1.00–1.051) in that age group, reflecting the importance of graft quality within the high-risk population. The results after LD transplantation were significantly better, a finding that is explained by favorable donor and recipient factors, such as shorter dialysis duration and cold ischemia, as well as younger donor age. Risk factors for death are donor type, recipient age, and presence of DM and IHD. Whereas risk factors for graft loss are IHD, donor type (DD/LD), and donor age. Finally, age was found to be a significant factor affecting graft and patient survivals in multifactorial regression analysis, within the older group of patients ≥70 years results after transplant were acceptable and not different to those of patients in the former decade of life.

## 5. Conclusions

Our results support continuous practice of the old-to old allocation policy based on utility considerations. However, whenever live donor transplantation is available, it should be encouraged. Transplant candidacy of elderly patients should be based on the patient’s general condition, performance, and cognitive status, as well as their predicted life expectancy. 

## Figures and Tables

**Table 1:** Characteristics of patients in the three groups: living donor, deceased donor old-to-old (DD-OTO), and deceased donor young-to-old (DD-YTO).

	Living Donor *n* = 257	DD Old-to-Old *n* = 213	DD Young-to-Old *n* = 123	*p* Value LD vs. DD	*p* Value OTO vs. YTO
Mean follow-up (months)	63.0 ± 49.5	59.4 ± 47.4	60.6 ± 49.2	0.714	0.082
Recipient age (years)	64.9 ± 3.8	65.7 ± 5.0	64.5 ± 3.6	0.036	0.012
Recipient Gender M/F (%)	76.4/23.1	78.4/21.6	69.6/30.4	0.180	0.480
Primary Disease (%)				0.014	0.133
HTN	10.1	15.5	9.6		
DM	32.2	32.4	20.0		
PCKD	12.4	12.7	11.2		
GN	7.4	7.5	12.8		
Pyelonephritis	3.9	2.3	5.6		
FSGS	4.3	7.0	7.2		
IgA	5.4	2.8	2.8		
Other	10.5	6.1	14.4		
Unknown	13.8	13.7	16.4		
Diabetes (%)	49.6	45.1	34.2	0.020	0.093
IHD (%)	32.9	39.7	42.2	0.159	0.770
PRA class I (%)	8.3	0.0	11.4	0.001	0.015
PRA class II (%)	6.6	0.0	3.9	0.002	0.016
Time on dialysis (mo.)	21.5 ± 22.7	63.3 ± 28.8	61.9 ± 33.8	*p* < 0.001	0.486
HLA-B full-match (%)	6.7	1.2	4.2	*p* < 0.001	0.273
HLA-DR full-match (%)	6.7	2.9	10.3	*p* < 0.001	0.058
Re-transplantation (%)	8.1	3.8	9.6	0.205	0.086
Donor age (years)	45.9 ± 12.4	65.9 ± 4.4	47.1 ± 11.0	*p* < 0.001	*p* < 0.001
Donor gender M/F (%)	54.3/45.7	56.6/43.4	60.5/39.5	0.521	0.730
Induction (%)				*p* < 0.001	0.828
IL-2 inhibitor	75.3	46.4	48.8		
ATG	8.6	48.8	45.5		
Desensitization (IVIG + PP + Rituximab)	8.7	0.0	0.0		
Cold ischemia time (hours)	3.5 ± 1.8	10.1 ± 3.7	10.8 ± 3.8	*p* < 0.001	0.524

DD, deceased donor; LD, living donor; OTO, old-to-old; YTO, Young-to-Old; HTN, hypertension; DM, diabetes mellitus; PCKD, polycystic kidney disease; GN, glomerulonephritis; FSGS, focal segmental glomerulosclerosis; IgA, immunoglobulin A; IHD, ischemic heart disease; PRA, panel reactive antibody, class I and II; HLA-B/DR match, human leukocyte antigen; IL-2 inhibitor, interleukin 2 inhibitor; ATG, anti-thymocyte globulin; IVIG, intravenous immune globulin; PP, plasmapheresis.

**Table 2:** Kidney transplant outcomes.

	Living Donor *n* = 257	DD Old-to-Old *n* = 213	DD Young-to-Old *n* = 123	*p* Value LD vs. DD	*p* Value OTO vs. YTO
DGF (%)	9.7	41.3	47.2	*p* < 0.001	0.255
PNF (%)	0.4	2.3	1.6	0.001	0.366
Graft Failure (%)	4.7	18.8	11.2	*p* < 0.001	0.531
Death (%)	15.5	36.2	40.8	*p* < 0.001	0.395
Death with functioning graft (%)	11.6	25.8	33.6	*p* < 0.001	0.066
Length of stay (days)	12.5 ± 23.7	15.7 ± 11.6	15.9 ± 12.2	0.081	0.201
Cr 30 days (mg/dL)	1.36 ± 0.67	2.90 ± 1.29	1.91 ± 1.28	*p* < 0.001	0.668
Cr 1 year (mg/dL)	1.22 ± 0.37	1.74 ± 1.12	1.79 ± 1.49	*p* < 0.001	0.717
Cr 5 years (mg/dL)	1.35 ± 1.14	2.29 ± 2.19	1.79 ± 1.49	*p* < 0.001	0.124

DD, deceased donor; LD, living donor; OTO, old-to-old; YTO, Young-to-Old; DGF, delayed graft function; PNF, primary nonfunction; Cr, creatinine.

**Table 3:** Cox regression multivariate analysis for death and graft loss.

**Risk Factors for Death**	**Hazard Ratio**	**95% Confidence Interval**	***p* Value**
Age	1.060	1.019–1.101	0.004
DM	1.773	1.241–2.532	0.002
IHD	1.510	1.063–2.145	0.021
Donor type (DD/LD)	2.865	1.910–3.800	*p* < 0.001
**Risk Factors for Graft Loss**			
IHD	1.782	1.045–3.038	0.034
Donor age	1.025	1.000–1.051	0.050
Donor type DD/LD	6.064	2.315–15.881	*p* < 0.001

DM, diabetes mellitus; IHD, ischemic Heart disease; DD, deceased donor; LD, living donor.

**Table 4:** Characteristics of patients in groups 60–69 and ≥70 years old.

	Patients 60–69 Years Old *n* = 493	Patients ≥ 70 Years Old *n* = 100	*p* Value
Recipient age (years)	63.7 ± 2.8	72.3 ± 2.4	*p* < 0.001
Recipient Gender M/F (%)	77.4/22.6	67/33	0.027
Primary Disease (%)			0.359
HTN	11.1	16.0	
DM	30.2	27.0	
PCKD	12.5	11.0	
GN	8.5	9.0	
Pyelonephritis	4.0	2.0	
FSGS	6.0	5.0	
IgA nephropathy	4.2	0.0	
Other	9.3	12.0	
Unknown	14.2	18.0	
Diabetes (%)	42.9	43.0	0.942
IHD (%)	38.0	33.3	0.392
PRA class I (%)	8.0	5.3	0.355
PRA class II (%)	3.7	3.7	1.000
Time on dialysis (mo.)	52.5 ± 31.3	46.4 ± 34.8	0.115
HLA-B full-match (%)	5.3	0.0	0.013
HLA-DR full-match (%)	7.0	2.6	0.108
Re-transplantation (%)	7.3	5.1	0.558
Donor age (years)	52.4 ± 13.8	57.8 ± 12.2	*p* < 0.001
Donor gender M/F (%)	55.2/44.8	63/37	0.230
Donor type LD (%)	19.8	12.8	0.016
Induction (%)			0.129
IL-2 inhibitor	59.2	61.0	
ATG	29.5	36.0	
Desensitization	4.3	1.0	
(PP ± Rituximab)	7.0	2.0	
Cold ischemia time (hours)	10.0 ± 3.5	10.7 ± 4.1	0.263

HTN, hypertension; DM, diabetes mellitus; PCKD, polycystic kidney disease; GN, glomerulonephritis; FSGS, focal segmental glomerulosclerosis; IgA, immunoglobulin A; IHD, ischemic heart disease; PRA, panel reactive antibody, class I and II; HLA-B/DR match, human leukocyte antigen; IL-2 inhibitor, interleukin 2 inhibitor; ATG, anti-thymocyte globulin; PP, plasmapheresis.

**Table 5:** Outcomes of patients in groups 60–69 and ≥70 years old.

	Patients 60–69 Years Old *n* = 493	Patients ≥ 70 Years Old *n* = 100	*p* Value
DGF (%)	28.5	32.0	0.746
PNF (%)	1.4	1.0	0.672
Graft failure (%)	32.5	32.0	0.929
Death (%)	27.6	31.0	0.493
Death with functioning graft (%)	17.9	26.0	0.054
Length of stay (days)	14.4 ± 19.4	14.0 ± 9.2	0.847
Cr 30 days (mg/dL)	1.76 ± 1.18	1.55 ± 0.67	0.084
Cr 1 year (mg/dL)	1.55 ± 1.09	1.38 ± 0.38	0.068
Cr 5 years (mg/dL)	1.77 ± 1.75	1.61 ± 0.78	0.668

DGF, delayed graft function; PNF, primary nonfunction; Cr, creatinine.

## Data Availability

The datasets used and/or analyzed during the current study are available from the corresponding author upon reasonable request.
